# Lessons from a school-based vaccination response following a Diphtheria outbreak in eThekwini district, SA

**DOI:** 10.4102/sajid.v39i1.610

**Published:** 2024-08-23

**Authors:** Azipheli E. Ngongoma, Moherndran Archary

**Affiliations:** 1 Department of Paediatrics and Child Health, Faculty of Health Sciences, University of KwaZulu-Natal, Durban, South Africa; 2 Department of Paediatrics, King Edward VIII Hospital, Durban, South Africa

**Keywords:** diphtheria, outbreak, vaccination, school-based programme, challenges

## Abstract

Diphtheria is a life-threatening respiratory tract infection that causes outbreaks in susceptible populations. Between April and May 2018, an outbreak of diphtheria occurred in the eThekwini district. A school-based outbreak vaccination response was initiated to target vulnerable children and adolescents.

**Contribution:**

This study adds to the limited data describing a school-based vaccination in an outbreak response and highlights successes and challenges. School-based outbreak vaccination response can rapidly increase vaccine coverage; however, additional community engagement may be required in vaccine-hesitant populations.

## Introduction

Diphtheria is a vaccine-preventable disease caused by infection with a toxigenic form of *Corynebacterium diphtheriae,* resulting in respiratory (most commonly) or cutaneous symptoms. Respiratory diphtheria presents with an upper respiratory tract illness characterised by a sore throat, low-grade fever and adherent membrane to the pharynx, tonsils or larynx, resulting in life-threatening airway obstruction and toxin-mediated cardiac or neurological dysfunction.^1^

Between 1980 and 2014, 412 cases of diphtheria were recorded in South Africa, with the vast majority (>80%) occurring before 1990.^2^ Two linked cases of diphtheria may be sufficient to constitute an outbreak, as the disease is now a rare infectious disease in the vaccine era.^3^ In 2015, an outbreak of respiratory diphtheria in two KwaZulu-Natal (KZN) health districts resulted in 15 cases in individuals ranging in age from four to 15 years, with a 27% case fatality rate.^4^ Inadequate diphtheria immunisations were documented in nine out of 12 cases (75%) under 18 years of age. Subsequent outbreaks included two laboratory-confirmed cases in 2016 from KZN and four cases from the Western Cape Province (WC) the following year. Two laboratory-confirmed cases of diphtheria were diagnosed in South Africa (SA) in April 2023. These outbreaks have often happened during periods of low immunisation coverage, especially of the booster diphtheria doses given to children at 6 and 12 years of age.^2^

Vaccines are among the most effective prevention tools available against infectious diseases and their sequelae, with over 57 million deaths averted.^5^ However, the success of immunisation programmes depends on high acceptance and coverage rates to achieve herd immunity.^2^ Reducing the incidence of a vaccine-preventable disease often leads to public perception that the severity and susceptibility of the disease have decreased.^4^ The vaccination coverage for diphtheria among 1-year-olds in SA was 87% in 2022 for the first dose; however, it dropped to 85% for the third dose and 71.9% for the fourth dose at 18 months. Vaccine coverage for diphtheria booster doses drops to 17% and 16% at six and 12 years, respectively, resulting in a vulnerable adolescent and adult population.^6^ Rapidly increasing vaccine coverage following an outbreak can interrupt community transmission and contain the outbreak.

During the diphtheria outbreak, four cases (two laboratories confirmed, one probable, one suspect), including two deaths (one laboratory confirmed, one probable case) were reported from the eThekwini district between 18 April 2018 and 17 May 2018.

This study describes an outbreak vaccination campaign conducted in school-going children as part of public health interventions following a diphtheria outbreak in eThekwini Metropolitan Municipality in 2018. The outbreak response also included individual case management, contract tracing, chemoprophylaxis for contacts and community health promotion activities.

## Methodology

We retrospectively reviewed tally sheets, meeting notes and other data collected during the outbreak response and school-based vaccination campaign following a diphtheria outbreak in eThekwini Metropolitan Municipality in 2018. As part of the outbreak response, 26 schools in the affected and adjoining sub-districts were identified to roll out a vaccination campaign. Healthcare workers from the school health teams and the expanded immunisation programme at primary health clinics in the affected areas were allocated to the closest school based on their geographical location. The Department of Education, school principals, governing bodies and community leaders approved the school-based outbreak response. Consent forms were given to schoolteachers to distribute to students at least 3 days before the planned date of the vaccination campaign at the school. The vaccinations were conducted on a single day during school hours as decided by school administrators, with an additional mop-up day for students absent on the main vaccination day.

Paper-based tally sheets were used to collect data when administering vaccinations during the vaccination campaign. Data from these tally sheets, in addition to school characteristics, including the total number of students enrolled, educational staffing composition, location and characteristics, were collated by the study investigator. The investigator anonymised all data and entered it into a data-secure Excel spreadsheet. A post-outbreak team meeting was held on 31 July 2018 with all healthcare workers who participated in the vaccination campaign, and the investigator reviewed the meeting minutes.

All qualitative data analyses were performed using SAS 9.4 (SAS Institute, CARY NC) and SPSS version 24 (IBM CORP. released in 2016). Continuous variables were summarised as means ± standard deviations (s.d.). Medians and interquartile ranges interquartile range (IQR) were summarised using proportions and percentages (%). Proportions were compared using Pearson’s chi-square and Fisher’s exact tests as appropriate.

### Ethical considerations

The study was approved by the Biomedical Research Ethics Committee of the University of KwaZulu-Natal (BREC/00002723/2021) and the KwaZulu-Natal Department of Health (KZ_202302_009).

## Results

A total of 25 103 students between the ages of five and 19 years were enrolled in the 26 schools identified in the area surrounding the cases of diphtheria. The distribution of the schools included 20 schools in urban areas (77%) and six schools in rural areas (23%), with nine high schools (35%) and 17 primary schools (65%).

A total of 20 509 students were vaccinated during the campaign, with an overall mean vaccination coverage of 81%. The vaccine coverage ranged from 69% in the rural area of Inkangala to 97% in the urban area of Umkomaas. There was no difference in the vaccine coverage when comparing rural to urban schools (82% vs. 79%, *p* = 0.58). The lowest vaccine coverage was 42% in a single primary school.

**FIGURE 1:**
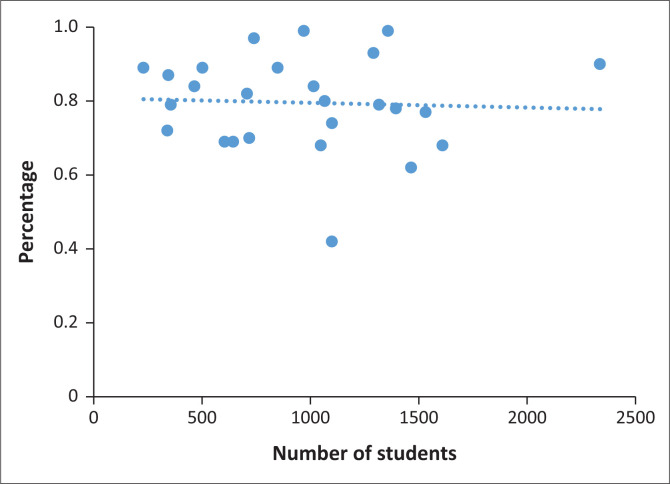
Percentage coverage by the size of the school.

Similarly, there was no significant difference in vaccine coverage by health facility conducting the campaign (*p* = 0.34) or when comparing high schools to primary schools (76% vs. 82%, *p* = 0.28) (Table 1). In addition, there was no significant correlation between the size of the school and vaccination coverage (*r* = −0.05) (Figure 1).

**TABLE 1:** Characteristics of schools included into the outbreak vaccination campaign.

Characteristic	*n*	Mean (%)	s.d.	*p*
**Location**				-
Amanzimtoti	1	89	-	-
Danyanga	2	89	0.00	-
Ifume	1	87	-	-
Inkangala	1	69	-	-
Isipingo	3	84	0.08	-
Lotus Park	1	74	-	-
Magabheni	4	75	0.05	-
Malukazi	1	77	-	-
Orient hills	2	71	0.40	-
Umgababa	2	84	0.22	-
Umkomaas	1	97	-	-
Umlazi	6	78	0.11	-
Winkelspruit	1	70	-	-
**Rural/urban**				0.58
Rural	6	82	0.12	-
Urban	20	79	0.13	-
**Health centre**				0.34
Danyanga	3	82	0.12	-
Isiphingo	4	74	0.24	-
Magabheni	5	74	0.05	-
Umlazi U21	9	80	0.10	-
Umnini	5	88	0.11	-
**Characteristics of school**				0.28
High school	9	76	0.09	-
Primary	17	82	0.14	-

s.d., standard deviation.

During the post-vaccination meeting, healthcare workers identified numerous challenges, particularly in schools with very low vaccine coverage. Obtaining consent from parents proved difficult, especially in schools with poor support for the vaccination campaign from teachers. Some parents refused vaccination for their children due to religious or other vaccine-related beliefs. Some specific challenges faced by healthcare workers included: not all learners returning consent forms, some learners refusing vaccination and learners opting to be vaccinated at clinics. Administrative challenges included staff shortages, lack of support and transportation issues for reaching schools without school health teams were also identified. Moreover, some principals refused to schedule additional dates for mop-up vaccinations to be conducted.

## Discussion

This school-based outbreak vaccination programme conducted over 2 weeks following a diphtheria outbreak in the eThekwini district resulted in a rapid increase in diphtheria vaccine coverage to 81%, contributing to the control of the outbreak. While there was no significant difference in coverage by type of school (rural vs. urban and primary vs. secondary), a few schools recorded very low coverage rates. These findings highlight the value of school-based vaccination campaigns during outbreaks.

Schools are an attractive site for delivering vaccines to children and adolescents because of the ability to reach many children quickly.^7^ For example, in April 2014, South Africa launched a countrywide human papillomavirus (HPV) vaccination campaign for Grade 4 girls aged nine. The campaign successfully immunised over 350 000 Grade 4 female students, reaching 94.6% of schools and 86.6% of age-eligible pupils in more than 16 000 public schools nationwide.^8^ School-based immunisation programmes have achieved high uptake and completion rates in several settings.^9,10,11,12,13,14,15^ These programmes have been shown to reduce sickness rates in vaccinated individuals and student household contacts, as seen with school-based influenza vaccine programmes.^12^

School-based vaccinations are associated with high satisfaction from various stakeholders, including school staff, immunisation nurses, public health professionals, teenagers and parents, who express satisfaction with this method. The convenience of school-based vaccination and the opportunity to receive peer support were key enabling factors.^9^ A study evaluating HPV vaccination found that community sensitisation meetings with parents and the lack of costs associated with vaccination were vaccine motivating.^16^ School exclusion during a pertussis outbreak was found to improve vaccine uptake but with a cost of lost days at school.^17^ Schools with vaccination clinics can significantly improve coverage and expedite the end of epidemics, as seen during a varicella outbreak.^10^ Continuous evaluation of vaccine coverage, safety and effectiveness, with feedback to stakeholders can also improve the efficiency and effectiveness of school vaccination programmes.^18^ During this study, the high vaccine coverage supports the use of schools to improve the rapid uptake of vaccinations.

School-based vaccinations can also face challenges, with school teachers responsible for the programme reporting feeling overworked.^9,19^ Obtaining parental approval for children to receive vaccinations remains the primary barrier to successful school immunisation. Recent programmes have seen children failing to return consent papers, partly because some parents were unfamiliar with the immunisation programmes and never received the necessary paperwork.^17,20,21,22^ Fear of side effects was a common reason for non-participation. However, increasing parental education has resulted in higher consent rates.^18,23^ Health professionals mentioned constraints in providing information, education and community resources such as posters or brochures due to time limitations, staff shortages, lack of water at schools and insufficient funds for local travel.

### Limitations

As the study was a retrospective review of data collected during the outbreak investigation, the data’s scope and completeness depended on the healthcare workers to ensure accuracy. A limited number (30) of healthcare workers were involved in the post-outbreak meeting.

## Conclusion

School-based outbreak vaccination programmes can rapidly achieve high coverage rates in rural and urban schools. Early coordination between the education and health departments and engagement with the community and adolescents will assist in vaccine uptake, especially in schools with low vaccine coverage. Maintaining high coverage of diphtheria booster vaccines in school-going children is essential to prevent future outbreaks.

### Recommendation

Including routine vaccinations as part of school health services can improve vaccine coverage, especially in early adolescents. Further studies should explore reasons for the non-uptake of vaccines and look at health worker challenges during the vaccine implementation.

Parental and adolescent education regarding routine immunisations is crucial to ensure acceptance of a school-based vaccine strategy.
